# Honeysuckle as a Bio-Enhancer in *Monascus purpureus* Fermentation: Synergistic Improvement of Monacolin K Yield and Flavor Complexity

**DOI:** 10.3390/foods15030560

**Published:** 2026-02-04

**Authors:** Arzugul Ablimit, Yike Zhai, Mengxue Chen, Qing Sun, Wenbo Liu, Duchen Zhai, Lichao Dong, Ang Huang, Baoguo Sun, Chengtao Wang, Chan Zhang

**Affiliations:** 1 Beijing Advanced Innovation Center for Food Nutrition and Human Health, School of Food and Health, Beijing Technology & Business University (BTBU), Beijing 100048, China; guzal_arzu@163.com (A.A.); 13014364618@163.com (Y.Z.); chenmengxue140116@163.com (M.C.); 13131535358@163.com (Q.S.); l18811301300@163.com (W.L.); duchenz0117@163.com (D.Z.); lichao_dong@163.com (L.D.); sunbg@btbu.edu.cn (B.S.); hongqumei@126.com (C.W.); 2 The First Medical Center, Chinese People’s Liberation Army General Hospital, Beijing 100853, China; huangangwin@aliyun.com

**Keywords:** fungal, co-fermentation, secondary metabolites, antioxidant activity, volatile flavor compounds

## Abstract

Recently, co-fermentation of functional medicinal plants with fungi has emerged as a promising strategy to enhance the overall quality of fermented foods. *Monascus* fermentation products have long been confronted with bottlenecks in both functionality and palatability, such as low monacolin K (MK) yield and poor flavor. Therefore, this study investigated the effects of co-fermenting *Monascus purpureus* with honeysuckle (*Lonicera japonica* Thunb.) on the bioactive metabolites and volatile flavor compounds of the fermented product. Through single-factor optimization, the addition of 0.8 g/L honeysuckle powder was identified as optimal, resulting in a 1.54-fold increase in MK yield compared to the control. Additionally, nine key genes were upregulated in the MK biosynthetic cluster (*mokA–mokI*). Co-fermentation also significantly increased the total flavonoid and polyphenol contents by 3.93-and 2.01-fold, respectively, and enhanced in vitro antioxidant activity. Gas chromatography-mass spectrometry analysis revealed that ketones, esters, and alcohols were the dominant volatile compounds. Orthogonal partial least squares-discriminant analysis identified 11 differential volatile compounds (variable importance in projection > 1), indicating a substantial shift in the flavor profile toward more desirable notes, with a reduction in undesirable aldehydes. These findings demonstrate that honeysuckle co-fermentation enhances the biofunctional properties of *M. purpureus* fermentation products and improves their sensory appeal, providing a viable bioprocessing strategy for developing high-value *Monascus*-based functional foods or ingredients.

## 1. Introduction

*Monascus purpureus* has long been used for traditional applications, including food preparation and healthcare. Owing to its numerous advantages, it has recently gained attention for both commercial and academic purposes [1]. The fermentation products of *M. purpureus* contain bioactive compounds such as monacolin K (MK), which have antibacterial [2], anticancer [3] and anti-inflammatory properties [4], and protect cardiovascular health [5]. The demand for *M. purpureus*-related products has steadily increased as interest in natural remedies and green health continues to grow. However, the *Monascus* industry still faces substantial challenges on its path toward high-value development. For instance, the low MK yield severely limits the potential for developing functional products. Simultaneously, traditional *Monascus* fermentation products exhibit certain shortcomings in flavor, further affecting their palatability and market acceptance. Current strategies for improving the flavor of *M. purpureus*, such as optimizing fermentation temperature, adjusting carbon-nitrogen ratios, or adding single flavor precursors, have shown certain limitations. These methods often only reduce the intensity of off-flavors to a certain extent without addressing the underlying causes or may lead to a decline in functional metabolite production when modifying flavor [6]. This is primarily because these methods fail to simultaneously regulate the metabolic pathways underlying both off-flavor formation and bioactive compound synthesis. Therefore, increasing MK production to enhance the functional activity of *M. purpureus*, while optimizing its undesirable flavors, is crucial for advancing the development and application of functional *M. purpureus* strains and related lipid-lowering products.

Food flavors typically consist of nonvolatile compounds responsible for taste, and volatile compounds responsible for aroma. Taste is perceived through the gustatory system—e.g., sweetness and umami—whereas the olfactory system detects aroma, typically caused by the synergistic effects of multiple components, involving sulfur-containing molecules, octenyl compounds, aldehydes, ketones, esters, and heterocyclic compounds [7]. Fermentation alters the nutritional and flavor profiles of food through the metabolic activity of microorganisms [8].

Traditional fermentation methods have certain limitations in enhancing secondary metabolite production in *M. purpureus*: the processing parameters are difficult to optimize for breakthroughs, and imprecise control makes the yield susceptible to raw material batch variations, resulting in instability and poor reproducibility [9]. Bidirectional fermentation, a novel bioengineering technology, uses specific microorganisms or enzymes to biotransform and modify traditional Chinese medicine (TCM) through interactions between microorganisms and TCM [10,11]. Compared to traditional fermentation, bidirectional fermentation is more cost-effective and is less influenced by environmental factors. It converts large molecular active substances in TCM into small molecules or vice versa, thereby effectively using these natural resources and enhancing their biological activities and functions [12,13].

Recently, co-fermentation of functional medicinal plants with fungi has emerged as a promising strategy to enhance the overall quality of fermented foods. In fermentation processes, *M. purpureus* serves as a key microorganism, facilitating the production and conversion of bioactive substances [11]. However, the selection of medicinal plants for co-fermentation requires precise matching with the metabolic characteristics of the target fungus, as not all plant additives can synergistically improve both functional metabolite production and flavor profiles. Honeysuckle (*Lonicera japonica* Thunb.), a TCM, is rich in chlorogenic acid, caffeic acid, and other phenolic compounds, which not only serve as direct precursors for enhancing antioxidant activity but also regulate the fermentation environment pH [14]. Unlike other medicinal plants containing complex and potentially inhibitory components, honeysuckle provides a balanced proportion of carbohydrates, proteins, and amino acids, which serve as carbon and nitrogen sources to promote mycelial growth and secondary metabolism of *M. purpureus* without inducing metabolic stress [15]. Honeysuckle itself contains characteristic volatile compounds, and its distinctive aroma profile has also become a key indicator for species identification and quality assessment, gaining increasing popularity among consumers worldwide [16,17]. However, research on its co-fermentation with *M. purpureus* remains limited, particularly regarding the synergistic mechanisms for enhancing MK production and improving flavor complexity. Therefore, this study investigates the effects of co-fermentation of *M. purpureus* with honeysuckle on the bioactive metabolites and volatile flavor compounds in the fermentation products.

Therefore, this study aims to comprehensively investigate the synergistic potential of co-fermentation involving honeysuckle and *M. purpureus*. By integrating fermentation optimization, metabolomic analysis, transcriptional profiling of MK biosynthetic gene clusters, and dynamic characterization of volatile flavor compounds via headspace solid-phase microextraction coupled with gas chromatography–mass spectrometry, we seek to elucidate the mechanistic basis for the production of *M. purpureus* secondary metabolites and the development of flavor profiles. This research provides a novel strategy for simultaneously enhancing both functional and sensory qualities of *M. purpureus* fermented products, thereby laying a solid foundation for developing a new generation of health-promoting functional foods.

## 2. Materials and Methods

### 2.1. Strain and Reagents

The wild-type *Monascus purpureus* M1 (CGMCC 3.0568), which exhibits a low yield of citrinin, is preserved by the Beijing Food Additive Engineering Technology Center.

The traditional Chinese medicine honeysuckle was purchased from Tong Ren Tang in Beijing. PDA medium, glucose, sucrose, rice flour, soybean flour, glycerol, peptone, KH_2_PO_4_, NaNO_3_, MgSO_4_·7H_2_O, and ZnSO_4·_7H_2_O were purchased from Beijing Jinlis Science and Technology Co., Ltd. (Beijing, China). Chromatographic-grade methanol and analytical-grade ethanol were purchased from Nanjing Shuner Biotechnology Co., Ltd. (Nanjing, China). The RNAprep Pure Polysaccharide and Polyphenol Plant Total RNA Extraction Kit, PrimeScript™ RT Reagent Kit, Reagent Enhanced Edition (SYBR Green) kit, FastQuant cDNA First Strand Synthesis Kit, and SuperReal PreMix Plus Kit were purchased from Tengen Biochemical Technology Company (Beijing, China). The total antioxidant Capacity (T-AOC) test kit (FRAP microplate method) was purchased from Shanghai Yuanye Biotechnology Co., Ltd. (Shanghai, China). The BCA protein concentration determination kit and the ABTS free radical scavenging ability detection kit were purchased from Beijing Solabao Technology Co., Ltd. (Beijing, China).

### 2.2. Single Factor Optimization Experiment

Upon activation of the strain on PDA medium at 30 °C for two consecutive generations, a spore suspension was then prepared. The seed culture medium and fermentation medium were prepared in accordance with the methods described by Zhang et al. [18]. After the seed liquid was expanded and cultivated, it was cultured in the fermentation broths of both the experimental group and the control group for a period of 15 days. Samples were aseptically collected at defined intervals (0, 3, 5, 8, 12, and 15 d) for subsequent analysis. To evaluate the effect of honeysuckle, three distinct treatments were established:

CK (Blank Control): Uninoculated, sterile fermentation medium.

MC (*Monascus* Control): *M. purpureus* cultivated in the base fermentation medium.

HS-MC (Honeysuckle-*Monascus* Co-fermentation): *M. purpureus* cultivated in the base fermentation medium supplemented with the optimal addition amount of honeysuckle powder.

Carry out a single-factor experiment regarding the liquid fermentation of honeysuckle, taking into account factors such as its form, concentration, and addition time, with the aim of ascertaining the optimal addition conditions.

For the decoction preparation: Firstly, weigh 5 g of honeysuckle, rinse it with clean water, and then soak it in 500 mL of distilled water for 1 h. Subsequently, heat it and maintain boiling for 30 min, followed by filtering out the juice. Repeat the above procedures, combine the two decocted medicinal liquids, add distilled water until the volume reaches 1000 mL, sterilize at 121 °C for 15 min, and reserve it for subsequent utilization.

Grinding of medicinal powder: Weigh a certain amount of honeysuckle, wash it with water, and let it air dry naturally. Use a grinding machine to grind it into powder and sieve it through an 80 mesh sieve. Sterilize it at 121 °C for 15 min for later use.

Add 1 mL, 2 mL, 4 mL, 6 mL, 8 mL, and 10 mL (volume fractions of 0%, 2%, 4%, 8%, 12%, 16%, and 20%, respectively) of honeysuckle juice and 0 g, 0.2 g, 0.4 g, 0.6 g, 0.8 g, and 1.0 g of honeysuckle powder to the liquid fermentation medium, and determine the content of MK in the fermentation broth. Based on the experimental results, determine the optimal form and amount of honeysuckle addition.

### 2.3. Fermentation Broth pH

A pH meter was used to measure the pH of the fermentation broth in the control and experimental groups at different fermentation stages.

### 2.4. Mycelial Biomass Assessment

The mycelial biomass was determined using the dry weight method. The fermentation broth (3 mL) was filtered through a sterilized filter paper and washed three times with distilled water to remove the culture medium components. After filtering out the water, it was dried in a 60 °C oven until reaching a constant weight. The filter paper containing mycelium was weighed again, and the difference between the two weights was considered the dry matter weight of the mycelium to be used in the following formula [19]:

Mycelial biomass (g/L) = dry matter weight/fermentation broth volume(1)

### 2.5. Residual Sugar Content

The total sugar content in the fermentation broth was determined using the phenol-sulfuric acid method. A glucose standard curve was prepared, and the sugar content in the fermentation broth was calculated based on the dilution ratio.

### 2.6. Protein Concentration

For sample processing, 1 mL of *M. purpureus* fermentation broth was added to 9 mL of 1×phosphate-buffered saline, extracted by ultrasound for 30 min, and centrifuged at 10,000 rpm for 10 min at 37 °C. The supernatant was collected, and the protein concentration was determined using a test kit.

### 2.7. Mycelial Morphology

The fermentation broth was resuspended at 4 °C, and a 2.5% glutaraldehyde solution was added to resuspend and fix the fungal mycelia in the dark, which were then maintained for 12 h. After graded dehydration using ethanol solutions from low to high concentrations, the residual ethanol in the centrifuge tube and thallus body was replaced with pure isoamyl acetate solution. A certain volume of hexamethyldisilazine solvent was added to completely cover the mycelium inside the centrifuge tube. The mycelium was sealed with degreased cotton and dried at 60 °C. The microstructures of different groups of mycelia were observed at different magnifications using scanning electron microscopy.

### 2.8. MK

The samples were pretreated before measurement of MK; 15 mL of 75% chromatographic methanol was added to 10% fermentation liquid and diluted 4 times using ultrasonic extraction for 30 min, and the supernatant was placed in darkness overnight. The supernatant was filtered with a 0.45 μm organic filter and set aside.

The production of MK was then measured using high performance liquid chromatography (HPLC), with an InertsilODS-3 C18 (150 mm × 4.6 mm × 5 μm) chromatography column, under the following conditions: mobile phase, B:A = 0.1% phosphoric acid: methanol = 1:3 (*v*/*v*); flow rate, 1 mL/min; detector, UV detector (PDA); detection wavelength, 237 nm; detector temperature, 30 °C; and sample volume, 10 μL [20].

### 2.9. Monascus Pigments

After adding 8 mL of ethanol solution (*v*/*v*, 70:30) to 1 mL of fermentation broth, the mixture was kept in a water bath at 60 °C for 1 h. After cooling, the solution was centrifuged at 4000 rpm for 15 min and kept in the dark until testing. The absorbance of the sample solution was measured using a UV spectrophotometer at maximum absorption wavelengths of 410, 448, and 505 nm for *Monascus* yellow, orange, and red pigments, respectively. The data were processed using the following formula [21]:

*Monascus* pigment color value (U/mL) = absorbance × dilution ratio (2)

### 2.10. Analysis of MK Synthesis-Related Genes at the Transcriptional Level

The fermented liquor was collected, transferred to a 2 mL centrifuge tube, and centrifuged at 13,780× *g* for 10 min. The supernatant was discarded and washed with sterile water, and the process was repeated four times. RNA was then extracted using a polysaccharide polyphenol plant total RNA extraction kit, according to the manufacturer’s instructions [22].

RNA quality and purity were assessed using a NanoDrop 2000 spectrophotometer (Thermo Fisher Scientific, Waltham, MA, USA) by detecting the absorbance ratios of A_260_/A_280_ and A_260_/A_230_. Samples with A_260_/A_280_ values between 1.8–2.0 and A_260_/A_230_ values ≥1.8 were considered to have high purity and were used for subsequent experiments. Additionally, 1% agarose gel electrophoresis was performed to verify the integrity of RNA.

cDNA synthesis was carried out using the PrimeScript™ RT Reagent Kit (Tengen Biochemical Technology Company) with gDNA Eraser to eliminate genomic DNA contamination. The reaction system (20 μL) included 2 μL 5×gDNA Eraser Buffer, 1 μL gDNA Eraser, 1 μg total RNA, and RNase-free ddH_2_O up to 10 μL. The mixture was incubated at 42 °C for 2 min to remove genomic DNA. Subsequently, 4 μL 5× PrimeScript Buffer 2, 1 μL PrimeScript RT Enzyme Mix I, 1 μL RT Primer Mix, and 4 μL RNase-free ddH_2_O were added to the system. The reverse transcription reaction was performed at 37 °C for 15 min, followed by inactivation at 85 °C for 5 s. The synthesized cDNA was stored at −20 °C for fluorescence quantitative PCR analysis.

*GAPDH* was selected as the internal reference gene, and the nine key sequences *mokA* to *mokI* related to MK synthesis in *M. purpureus* provided by the National Center for Biotechnology Information (NCBI) website were selected as target genes (Table S1). The samples were processed according to the instructions of the SuperReal Fluorescence Quantitative Premixed Reagent Enhanced Edition (SYBR Green) kit (Tengen Biochemical Technology Company).

### 2.11. Detection of the Total Polyphenol Content

After placing 100 μL of the sample into a centrifuge tube, 50 μL of Folin phenol reagent was added, mixed well, and reacted at room temperature for 5 min. Afterward, 150 μL of 20% Na_2_CO_3_ solution was added, mixed well, and reacted for 20 min. Then, the absorbance at 765 nm was measured. Using the same method, a standard curve was prepared using gallic acid as the standard, and the total polyphenol content of each fermentation broth was calculated based on this curve [23].

### 2.12. Detection of the Total Flavonoid Content

The aluminum nitrate colorimetric method was used to determine the total flavonoid content in different groups of fermentation broths. The reaction solution was prepared in a ratio of 1:1.5:1.5 as follows: sample solution, 5% NaNO_2_ solution, 10% Al(NO_3_)_3_ solution, respectively. After each addition, the solution was mixed well and kept in the dark for 5 min. Finally, 2 mL of 4% NaOH solution was added, and the solution was kept for 15 min before measuring the absorbance at 510 nm. Using the same method, a standard curve was prepared with rutin as the standard, and the total flavonoid content in different groups of fermentation broth was calculated based on this curve [24].

### 2.13. Detection of Scavenging Activityof Superoxide Anion Radical

By transferring 200 μL of fermentation extract separately into 2 mL centrifuge tubes, the blank, control, measurement, and positive control groups were established, with at least three replicates per group. Distilled water was used as the blank control, and a vitamin C solution (0.5 mg/mL) was selected as the positive control. After measuring the absorbance of the reaction solution at 320 nm, the superoxide anion radical scavenging activity is calculated according to the following formula:

(3) $$free\;radical\;scavenging\;rate\;(\%)=\frac{A_{blank}-\left(A_{measurement}-A_{control}\right)\;}{A_{blank}}$$

### 2.14. Detection of the Free Radical-Scavenging Activity Using the ABTS Assay

Using the method described by Qi et al. [25] with slight modifications, we conducted experiments using the 2,2′-azino-bis-3-ethylbenzthiazoline-6-sulphonic acid (ABTS) kit from Beijing Solaibao Technology Co., Ltd. (Beijing, China) according to the manufacturer’s instructions.

### 2.15. HS-SPME-GC–MS Sample Extraction

Solid-state fermentation: Dried honeysuckle was ground and passed through a 40-mesh sieve. The honeysuckle powder was mixed with distilled water at a solid-to-liquid ratio of 1:1 (g:mL) in a 250 mL conical flask and soaked at room temperature for 2 h. The mixture was then sterilized at 121 °C for 20 min to obtain the solid honeysuckle culture medium. *M. purpureus* spores (1 × 10^6^) were inoculated into the medium and statically cultured at a constant temperature of 30 °C for 15 days [26].

Take 1–2 g of the solid-state fermentation sample and add an appropriate amount of ultrapure water to prepare a homogenate. Transfer the sample into a headspace solid-phase microextraction (HS-SPME) vial, followed by the addition of 1 g of NaCl and 10 μL (50 μg/mL) 2-octanol (internal standard solution). The mixture was equilibrated at 40 °C with agitation for 20 min using the GC-MS heating module. Volatile compounds were then extracted using an SPME fiber for 30 min, followed by thermal desorption for 5 min.

### 2.16. GC–MS Conditions

An HP-5MS UI capillary column was used. The temperature program was set as follows: initial hold at 40 °C for 3 min; increase to 120 °C at 5 °C/min and hold for 3 min; ramp to 230 °C at 20 °C/min and hold for 5 min; finally, increase to 250 °C at 8 °C/min and hold for 5 min. The inlet temperature was set at 250 °C, with helium (He) as the carrier gas at a flow rate of 1.0 mL/min in splitless mode. The solvent delay was set to 1 min.

Electron impact (EI) ionization was used with an electron energy of 70 eV. The transfer line and ion source temperatures were both set at 230 °C, and the quadrupole temperature at 150 °C. Mass spectra were acquired over a scan range of 35–500 *m*/*z*.

### 2.17. Data Processing

All experiments were performed in triplicate (*n* = 3), and data are presented as mean ± standard deviation (SD). Statistical analysis was conducted using SPSS Statistics 27.0. One-way analysis of variance (ANOVA) was employed to assess the significance of differences among treatment groups. Significance levels are denoted in the figures as follows: * *p* < 0.05, ** *p* < 0.01, or with different lowercase letters (e.g., a, b, c). Principal component analysis (PCA) and orthogonal partial least squares discriminant analysis (OPLS-DA) were performed using SIMCA14.1.

## 3. Results and Discussion

### 3.1. Single-Factor Optimization of Adding Honeysuckle to M. purpureus Cultures

The addition of certain exogenous substances is closely associated with the growth of microorganisms [27]. Therefore, honeysuckle underwent systematic pretreatment to prepare two forms of additives for fermentation: juice and powder. Thallus biomass and MK production were used as indicators to evaluate the effects of different forms and addition levels of honeysuckle on *M. purpureus* growth and secondary metabolism. It was observed that there were significant differences in the effects between honeysuckle juice and powder: the promotion of biomass by juice was not completely positively correlated with its addition amount, and inhibitory effects were exhibited at higher concentrations. In contrast, honeysuckle powder consistently promoted thallus growth and significantly increased MK yield.

**Figure 1:**
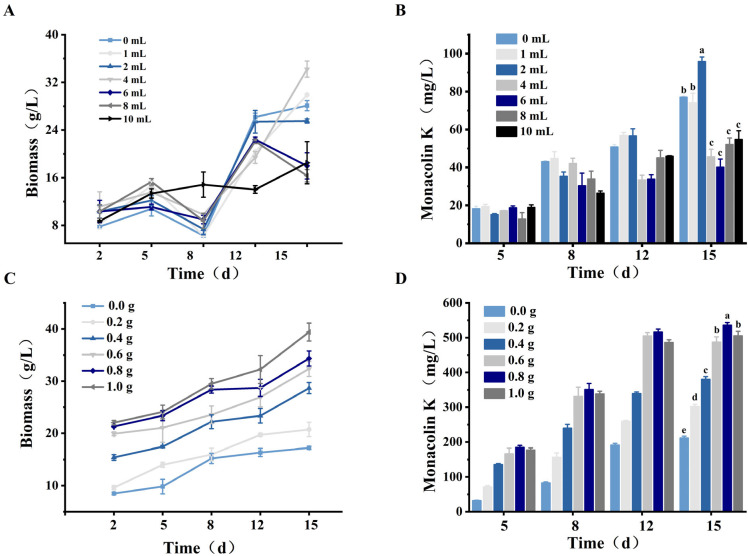
Influence of the addition form and amount of honeysuckle on *M. purpureus*: (**A**) Effect of juice concentration on biomass. (**B**) Effect of juice concentration on MK yield. (**C**) Effect of powder concentration on biomass. (**D**) Effect of powder concentration on MK yield. Note: Different lowercase letters indicate a statistically significant difference (*p* < 0.05).

As shown in Figure 1, the thallus biomass showed an upward trend with increasing amounts of honeysuckle powder. The highest level (1.0 g) exhibited the most pronounced biomass-promoting effect, reaching a maximum of 39.42 mg/L; this was 1.29 times higher than that of the control (CK) group. The observed increase in biomass and thallus density might have influenced the oxygen transfer dynamics within the limited fermentation space, which could be a contributing factor to the altered metabolite profile. Therefore, the amount of honeysuckle added was not absolutely correlated with secondary metabolite production. The addition of 0.8 g of honeysuckle powder had the strongest promoting effect on MK synthesis at each time point. On the 15th d of fermentation, the yield reached 536.10 mg/L, which was nearly 1.54 times higher than that of the CK group (*p* < 0.05). Therefore, based on the changes in MK concentration and thallus growth, 0.8 g of honeysuckle powder was used in all subsequent experiments.

### 3.2. Effects of Honeysuckle on the Growth of M. purpureus

**Figure 2:**
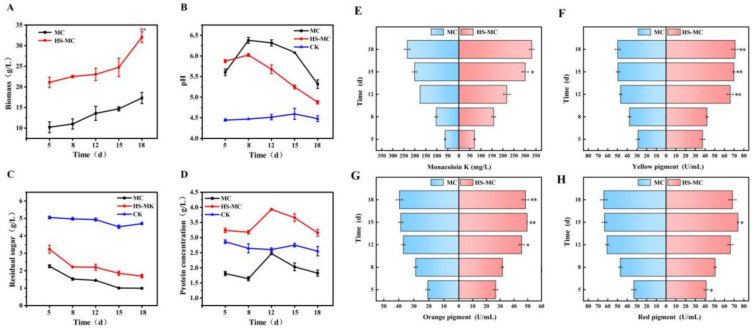
Effects of honeysuckle on the growth and metabolism of *M. purpureus*: (**A**) Biomass. (**B**) pH of fermentation fluid. (**C**) Residual sugar concentration. (**D**) Protein concentration. (**E**) MK. (**F**) Yellow pigment. (**G**) Orange pigment. (**H**) Red pigment. Note: ** *p* < 0.01, represents the significant difference between the experimental group and the control group, * represents *p* < 0.05.

Based on the optimized conditions for adding honeysuckle, samples were collected at different time points of fermentation to measure growth indicators. The addition of honeysuckle increased the total sugar concentration in the fermentation broth, thereby providing a carbon source for *M. purpureus* (Figure 2C). Although the residual sugar content in the fermentation broth of the honeysuckle–*M. purpureus* co-fermentation (HS-MC) group was relatively high, sugar consumption increased by 38% compared to that of the *M. purpureus* control (MC) group during the early growth stage (*p* < 0.05). Following the addition of honeysuckle to the *M. purpureus* culture, the material utilization rate greatly improved, thallus growth rate accelerated; on the 5th d of fermentation, biomass increased 1.07 times higher than that of the MC group, accompanied by a notable increase in thallus density. The HS-MC group also exhibited improved growth throughout the entire fermentation cycle; even in the late stages of fermentation, compared to the MC group, biomass was increased by 85% (*p* < 0.01) (Figure 2A).

Honeysuckle contains nutrients such as proteins, fat, and vitamins, as well as various amino acids, including aspartic acid, lysine, glutamic acid, and leucine, promoting thallus growth and metabolism [28]. Notably, honeysuckle provides nitrogen for *M. purpureus*. *M. purpureus* also utilizes the energy-rich metabolites of honeysuckle, thereby accelerating metabolism in the thallus body. The promotion of primary metabolites, such as amylase, consistently increased the protein concentration in the fermentation broth compared to that in normal culture medium. The protein concentration of the HS-MC group reached 3.93 mg/mL on the 12th d of fermentation, showing increases of 59.1% and 51.1% compared with that of the MC and CK groups, respectively (*p* < 0.05) (Figure 2D). In the later stages of thallus fermentation, secondary metabolism is primarily used, and proteins are mostly used for thallus growth and secondary metabolite synthesis; therefore, a downward trend was observed. As fermentation progressed, the pH value of the HS-MC and MC groups gradually decreased, with the HS-MC group exhibiting a more apparent decrease (Figure 2B). Additionally, chlorogenic and caffeic acids, which are abundant in honeysuckle, created a lower pH fermentation environment for *M. purpureus*, which promoted *M. purpureus* growth and secondary metabolite synthesis [29,30].

### 3.3. Effects of Honeysuckle on the Metabolism of M. purpureus

Under initial fermentation conditions with a low pH, the ability of *M. purpureus* to synthesize yellow and orange pigments was greatly enhanced [31]. Therefore, based on the pH results, the effects of honeysuckle on secondary metabolites synthesis by *M. purpureus* were investigated (Figure 2E). It promoted the synthesis of secondary metabolites by *M. purpureus*, with the greatest impact being on MK synthesis. The MK yield of the HS-MC group remained consistently higher than that of the MC group, with a significant difference on the 15th d (*p* < 0.05). Due to the continuous synthesis and extracellular secretion of MK in the late fermentation stage, the highest extracellular MK yield was achieved after 18 d of fermentation, reaching 330.90 mg/L, a 41.5% increase compared to the yield of the MC group. In a previous study, after solid-state fermentation with *M. purpureus*, TCM increased the production of MK and promoted the biotransformation of active ingredients in TCM, which has significant implications for the development and application of *M. purpureus* TCM combined with lipid-lowering drugs [32].

The changes in the three types of *Monascus* pigments exhibited consistent trends with a significant effect on the increase in yellow pigment production during the late stages of fermentation (*p* < 0.01). On the 18th d, the concentration of this pigment reached 71.04 U/mL, representing a 42.8% increase compared with that in the MC group (Figure 2). The concentration of the red pigment also increased; however, this change was not significant. The promoting effect of honeysuckle was most pronounced in the early stages of fermentation, resulting in a 24.3% increase in concentration. During the synthesis of *Monascus* pigments, the starting substrate undergoes polymerization and transesterification reactions to produce orange pigments, which are then converted into red and yellow pigments through ammonification and reduction reactions, respectively [33]. The addition of honeysuckle enhances the reducibility of the fermentation environment, further affecting relevant reactions inside the thallus. An increase in reducible substances promotes the production of yellow pigments through reduction reactions. In addition, amino acids enriched in honeysuckle positively impacted the synthesis of *Monascus* pigments and increased pigment production [34].

**Figure 3:**
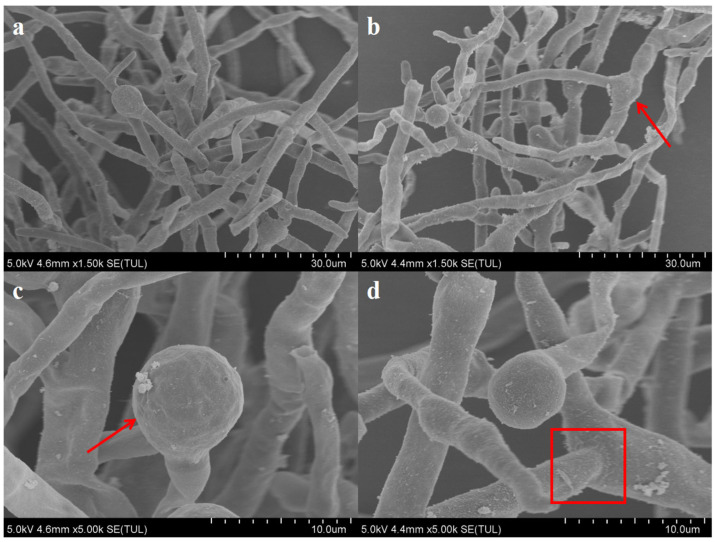

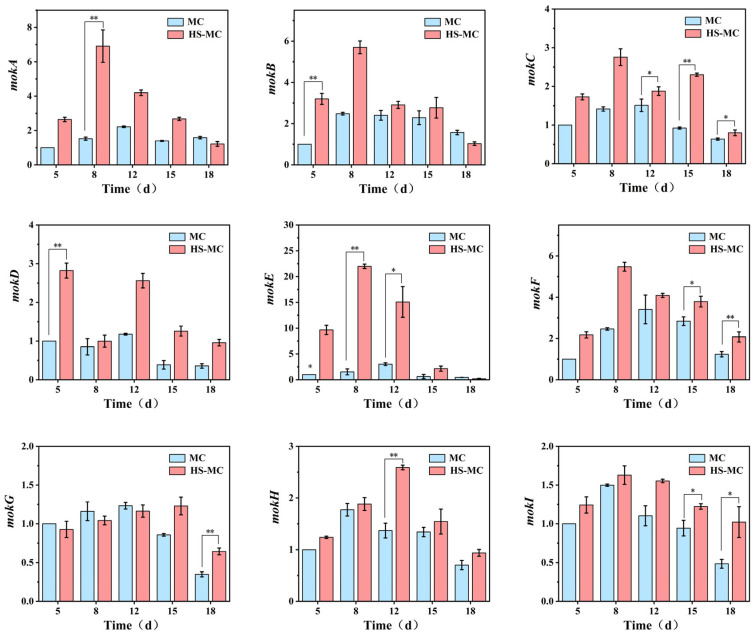
Effects of honeysuckle addition on mycelium morphology (**upper panel**) and gene expression related to MK synthesis in *M. purpureus* (**lower panel**): (**a**) MC—1500×. (**b**) HS-MC—1500×. (**c**) MC—5000×. (**d**) HS-MC—5000×. Note: ** *p* < 0.01, represents the significant difference between the experimental group and the control group, * represents *p* < 0.05.

The expression levels of the nine key genes (*mokA–mokI*) in their synthesis gene cluster were analyzed to further explore the potential regulatory role of honeysuckle in MK synthesis (Figure 3). Honeysuckle addition led to the upregulation of all nine key genes related to MK synthesis at the transcriptional level, though the fold changes varied and were relatively modest for most genes. The transcription levels of *mokA, mokC*, and *mokE* showed statistically significant changes during the early stages of fermentation (*p* < 0.05). Among these, *mokE* exhibited the most prominent change on the 8th d of fermentation, with a transcriptional level increase of 13.4% compared to the MC group, while *mokA* and *mokC* levels increased by 3.54% and 0.94%, respectively.

*mokA* and *mokE* are primarily involved in encoding polyketide synthase, a key enzyme of *M. purpureus*, for the synthesis of three secondary metabolites, *Monascus* pigments, MK, and citrin. *mokB* catalyzes the synthesis of the diketone portion of the MK side chain and is associated with the upregulation of early gene expression, with all three genes playing important roles in MK synthesis [35]. *mokH* does not directly participate in MK synthesis, but it encodes the Zn(II) 2Cys6 binuclear DNA-binding protein, which activates MK synthesis [36]. Zhang et al. demonstrated that *mokH* overexpression increased MK production by 82% [37], suggesting that even moderate changes in the transcription of regulatory genes like *mokH* may have functional relevance. In our study, *mokC* expression did not show a statistically significant change, while *mokG* expression in the HS-MC group was slightly downregulated. Notably, the protein encoded by *mok*I contributes to substrate efflux; its transcription level increased in the late stages of fermentation in the presence of honeysuckle, which may facilitate the extracellular transport of MK and reduce its feedback inhibition [38]—this provides a potential supplementary mechanism for MK accumulation beyond de novo synthesis.

It should be emphasized that the observed transcriptional changes are correlative rather than definitively causal for the significant increase in MK yield. These transcriptional upregulations may contribute to MK accumulation by creating a favorable metabolic environment for MK synthesis, but they are unlikely to be the sole mechanistic driver. Instead, the synergistic enhancement of MK yield is proposed to result from multiple interconnected factors: the transcriptional upregulation of key biosynthetic genes (providing a transcriptional basis for increased enzyme synthesis), the improved nutritional supply and metabolic efficiency (enhancing precursor availability), the optimized fermentation microenvironment (promoting metabolic activity), and the enhanced MK efflux (reducing feedback inhibition).

Considering that adding honeysuckle significantly promoted the growth and overall density of the thallus, thallus morphology in the various experimental groups was assessed using scanning electron microscopy (Figure 3). Observing the overall state of the mycelium at low magnification, the addition of honeysuckle increased the mycelial density; however, the mycelial tightness was lower in the HS-MC group than in the MC group. The mycelium of the HS-MC group was relatively independent and unaffected by mutual entanglement, which did not impact growth and metabolism. Scanning electron microscopy observations showed that the mycelia of the HS-MC group were relatively independent with increased inter-thallus gaps, which we hypothesize may enhance cell membrane permeability. This potential increase in permeability could facilitate the secretion of synthesized products such as MK, thereby reducing feedback inhibition and promoting further synthesis. While direct measurements of membrane permeability were not performed in this study, this hypothesis is supported by the consistent upregulation of *mok*I (a gene involved in MK efflux) and the significantly higher extracellular MK yield in the HS-MC group. The graph shows that the thallus surfaces in the HS-MC group accumulated more secreted substances. Observing thallus morphology at high magnification revealed that, compared to the MC group, the spore heads and surfaces of the thallus in the HS-MC group were relatively smooth, without detectable indentation or wrinkling, and little individual difference was noted in the mycelia. The morphology of the fungal hyphae affected their biological properties, and changes occur under different fermentation conditions [39].

### 3.4. Effects of Honeysuckle on the In Vitro Antioxidant Activity of M. purpureus Fermentation Products

**Figure 4:**
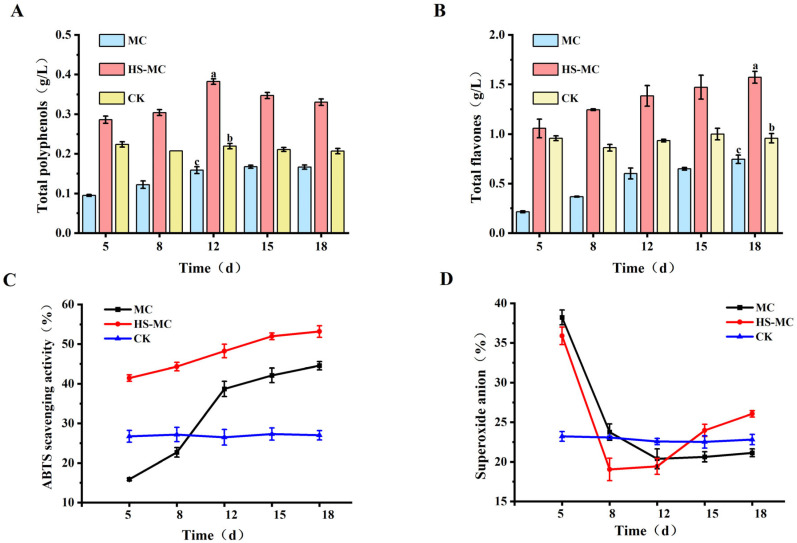
Effect of addition of honeysuckle on antioxidant activity in vitro: (**A**) Total polyphenols. (**B**) Total flavones. (**C**) ABTS scavenging activity. (**D**) Superoxide anion scavenging activity. Note: Different lowercase letters indicate a statistically significant difference (*p* < 0.05).

Microbial fermentation enhanced the antioxidant activity of natural products [40]. Considering the fermentation characteristics of *M. purpureus*, the addition of honeysuckle resulted in a significant increase in the total polyphenol and flavonoid contents of the co-fermented products, and the total amount showed an upward trend with prolonged fermentation time (Figure 4). The increases in total flavonoid and polyphenol levels in the early stages of fermentation were significant, with contents increasing by 3.93 and 2.01 times, respectively, compared with the levels in the MC group (*p* < 0.01). Honeysuckle addition elevated the content of these flavonoids and polyphenols or related components. However, their overall level in the HS-MC group was higher than that in the CK group, thereby *M. purpureus* fermentation contributed [41] to the production of β-glucosidase and other substances in its primary metabolism process. Therefore, the polyphenol and flavonoid content was enhanced in the fermentation broth [42].

The ability of the co-fermentation products of *M. purpureus* and honeysuckle to scavenge free radicals was also improved, as confirmed by the ABTS assay. The overall free radical level in the HS-MC group remained higher than that of the MC and CK groups, with a maximum increase of 1.61 times the level of the MC and CK groups (*p* < 0.05). The level of reactive oxygen species in an organism remains consistently in a state of dynamic balance. If this balance is disrupted, free radicals can easily cause damage and potentially exacerbate the development of atherosclerosis [43]. Therefore, substances with strong antioxidant capacity, such as superoxide dismutase, can prevent oxidative damage by reducing the level of reactive oxygen species, thereby preventing cardiovascular and cerebrovascular diseases [44]. In the current study, the co-fermentation of honeysuckle and *M. purpureus* enhanced the antioxidant activity of the synthesized products, providing possibilities for their application as a lipid-lowering drug.

### 3.5. Analysis of Volatile Flavor Compounds

**Figure 5:**
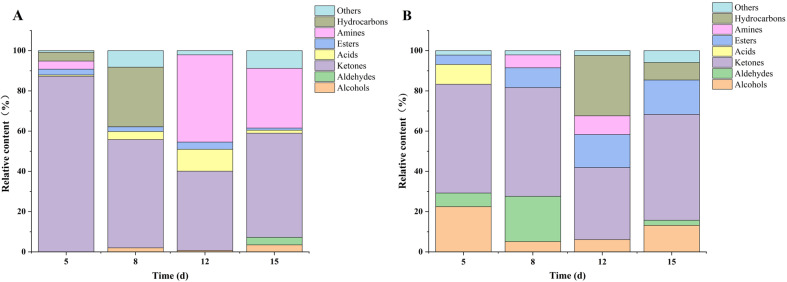
The changes in volatile flavor substances during the fermentation process. (**A**) MC. (**B**) HS-MC.

Gas chromatography-mass spectrometry was used to analyze volatile flavor compounds during the co-fermentation of honeysuckle and *M. purpureus*. Database matching identified various volatile compounds in both the MC (pure *M. purpureus*) and HS-MC groups at 5, 8, 12, and 15 d of fermentation. The relative content of different categories of volatile compounds varied across fermentation stages (Figure 5). Notably, the types and relative abundances differed significantly between the MC and HS-MC groups at each time point.

Fermentation technology modulates food matrices via microbial metabolic activities, dynamically altering nutritional profiles and flavor signatures. Empirical evidence suggests that fermentation impacted the production of esters and degradation of off-flavor compounds, thereby influencing the equilibrium between desirable fruity notes and mitigation of undesirable flavor [45].

Ketones were the most abundant volatile compounds in the experimental groups, followed by alcohols and esters. On day 15, ketones comprised 52.6% of total volatiles, whereas esters and alcohols accounted for 17.1% and 13.2%, respectively. These compounds contribute to flavor in *M. purpureus* and other mold-based fermentation processes. In the MC group, ketones also dominated, followed by amines and aldehydes. On the final day, ketones and amines accounted for 51.72% and 29.67%, respectively. Ketone production may be associated with polyketide synthase (PKS), an enzyme unique to *Monascus* that primarily catalyzes the biosynthesis of pigments and MK. PKS activity may also indirectly promote the formation of certain ketones via secondary metabolism of polyketide intermediates. Additionally, the β-oxidation of lipids or long-chain fatty acids by *Monascus* generates methyl ketones, imparting nutty, cheesy, and other characteristic flavors to the fermented products at low concentrations [46].

Alcohol levels increased rapidly in the early stages of co-fermentation, likely owing to microbial degradation of sugars and other substrates. However, the alcohol content declined in the mid-to-late stages, possibly due to esterification or other secondary metabolic reactions that converted alcohols into esters or aromatic compounds. This contributed to increased ester content observed during the later fermentation stages [47]. Co-fermentation also resulted in a reduction in undesirable aldehydes. Aldehydes are chemically unstable and may be reduced to alcohols or oxidized to acids through microbial activity [48]. As high aldehyde levels are correlated with lower consumer acceptance, their decline suggests that the co-fermentation of honeysuckle and *M. purpureus* may improve overall flavor by minimizing off-flavors. In the later fermentation stages, a notable increase in terpenes and esters transformed the flavor profile from a simple nutty note to a more complex bouquet with floral, fruity, and spicy characteristics.

**Figure 6:**
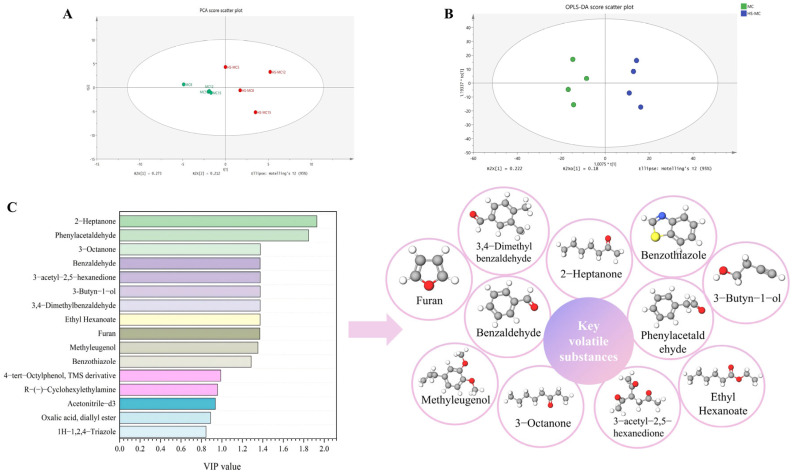
The analysis of volatile compounds. (**A**) PCA score scatter plot. (**B**) OPLS-DA score scatter plot. (**C**) VIP value of differential aroma compounds. Note: * indicates samples in the HS-MC group that exhibit significant separation from the MC group in the model (*p* < 0.05).

Unsupervised principal component analysis (PCA) was performed on samples at the end of fermentation to assess differences in volatile profiles between the two groups (Figure 6A). Two principal components were identified: PC1 explained 27.3% of the variance, and PC2 accounted for 21.2% of the variance. Within the 95% confidence interval, the MC group was separated from each co-fermented group, indicating a significant divergence in volatile profiles. As fermentation progressed, the samples from the MC and HS-MC groups became increasingly distinct, with volatile compounds at different fermentation stages being distributed across different PCA quadrants. This finding reflects the growing differences in both composition and concentration of volatile substances over time.

An orthogonal partial least squares-discriminant analysis (OPLS-DA) model was constructed to further investigate the differences in volatile flavor compounds between the two fermentation groups. The two groups were separated in the OPLS-DA score scatter plot (Figure 6B), indicating that the model effectively captured inter-group variation and distinguished volatile profiles from the different fermentation treatments.

Variable importance in projection (VIP) values were calculated to identify key contributors to these differences (Figure 6C). Eleven differential compounds with VIP > 1 were identified, including three ketones, one alcohol, one ester, three aldehydes, one phenol, and others (Table 1). These compounds included 3-butyn-1-ol, benzaldehyde, 3,4-dimethylbenzaldehyde, phenylacetaldehyde, furan, 3-acetyl-2,5-hexanedione, 3-octanone, 2-heptanone, ethyl hexanoate, methyleugenol, and benzothiazole.

The formation of flavor compounds is largely driven by complex microbial metabolism. Under natural conditions, microorganisms and endogenous enzymes convert sugars, proteins, and lipids into characteristic aroma compounds—including aldehydes, alcohols, ketones, and esters—via carbohydrate metabolism, protein hydrolysis, amino acid catabolism, and lipid degradation [26,49].

**Figure 7:**
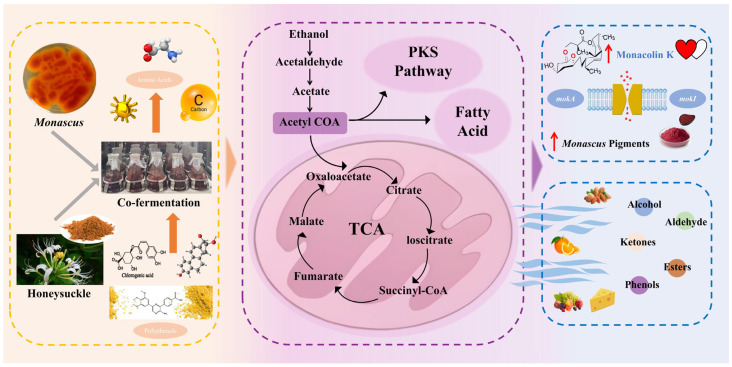
Proposed mechanism for the synergistic enhancement of MK yield and flavor complexity in *M. purpureus* via honeysuckle co-fermentation.

The abundant carbohydrates and amino acids provided by honeysuckle likely served as pivotal precursors, driving microbial metabolism through enhanced glycolysis and the tricarboxylic acid cycle. This accelerated primary metabolism, which generated a surplus of acetyl-CoA, the central precursor for both MK and pigment synthesis, as well as the production of various volatile compounds through fatty acid metabolism [1]. The significant increase in ketones, such as 3-octanone and 3-acetyl-2,5-hexanedione, can be attributed to the β-oxidation of fatty acids derived from fungal lipids or honeysuckle itself [50]. Notably, the PKS pathway may also contribute to the formation of certain methyl ketones through the degradation of polyketide chain intermediates [51]. The co-occurrence of enhanced MK production and ketone abundance supports this interconnected metabolic flux (Figure 7).

The conversion of aldehydes to alcohols and subsequently to esters represents a crucial pathway for enhancing flavor. The observed decline in aldehydes, followed by an initial surge, and then a decrease in alcohols, indicates active microbial transformation. Aldehydes, often associated with grassy or pungent off-flavors, can be reduced to their corresponding alcohols by alcohol dehydrogenases in *M. purpureus* [52]. Subsequently, these alcohols underwent esterification with acyl-CoAs (activated fatty acids) catalyzed by alcohol acyltransferases, leading to the formation of esters that impart fruity and floral notes [53]. Esters are particularly important aroma-active compounds formed through the degradation of fatty acids and esterification of acids and alcohols [54]. *M. purpureus* fermentation promoted ester production through such metabolic pathways [55,56]. The increased ester content observed in the co-fermentation of honeysuckle and *M. purpureus* likely enhanced the aroma complexity, as esters exhibit synergistic effects with other compounds to intensify the overall aromatic profile.

Furthermore, the metabolism of amino acids derived from the protein content of honeysuckle contributed to flavor complexity through the Ehrlich pathway [57]. This pathway involved the deamination and decarboxylation of amino acids such as leucine, valine, and phenylalanine into aldehydes, which are then reduced to alcohols or oxidized to acids. These products serve as direct precursors for esters and other complex volatiles [58]. The presence of benzeneacetaldehyde, which has a honey, floral descriptor, may originate from phenylalanine metabolism, highlighting the role of amino acid conversion in shaping the aroma profile.

In summary, the modified flavor profile in the co-fermentation of honeysuckle and *M. purpureus* is caused by a synergistic effect between the enhanced carbon flux, promoted fatty acid β-oxidation, and activated amino acid metabolism, coupled with efficient enzymatic transformations that steer the metabolic intermediates toward desirable aroma compounds. This proposed mechanism highlights the potential of using specific TCMs as a strategic tool to modulate microbial metabolic pathways, thereby simultaneously enhancing both the functional and sensory qualities of fermented foods. Future studies employing transcriptomic and enzymatic analyses are warranted to validate these hypothesized pathways at a molecular level.

## 4. Conclusions

This study demonstrated that honeysuckle co-fermentation serves as an effective bioprocessing strategy for simultaneously overcoming two major bottlenecks in *M. purpureus* products: low MK yield and undesirable flavor. Through single-factor optimization, 0.8 g/L honeysuckle powder was identified as the optimal addition condition, which significantly increased MK yield by 1.54-fold compared to the control, with upregulation of nine key genes (*mokA–mokI*) in the MK biosynthetic cluster. Honeysuckle co-fermentation synergistically enhanced the biofunctional properties of the product, increasing total flavonoid and polyphenol contents by 3.93-and 2.01-fold, respectively, and improving in vitro antioxidant activity. The flavor profile of the fermented product was substantially improved: GC-MS combined with multivariate statistical analysis identified 11 differential volatile compounds, with ketones, esters, and alcohols becoming dominant, and undesirable aldehydes reduced, leading to a more complex and appealing flavor.

This study presents a viable and innovative paradigm for developing the next generation of *Monascus*-based functional foods, where exceptional functionality and palatability are synergistically achieved. Future research will focus on clarifying the specific transformation pathways of flavor precursors using metabolomics technology to further optimize the quality of co-fermented products.

## Figures and Tables

**Table 1:** Key volatile flavor substances during fermentation.

Name	CAS	Formula	VIP_Value	Descriptor	Category
3-Butyn-1-ol	927-74-2	C_4_H_6_O	1.3746	Grass	Alcohol
3,4-Dimethylbenzaldehyde	5973-71-7	C_9_H_10_O	1.3738	Almond, cherries	Aldehydes
Benzaldehyde	100-52-7	C_7_H_6_O	1.3733	Bitter almond, cherry	
Phenylacetaldehyde	122-78-1	C_8_H_8_O	1.8472	Honey, flower	
Furan	110-00-9	C_4_H_4_O	1.3709	Baking, smoky	Furan derivative
3-Acetyl-2,5-hexanedione	42781-07-7	C_8_H_12_O_3_	1.3747	Sweet, burnt sugar	Ketones
3-Octanone	106-68-3	C_8_H_16_O	1.3748	Milk, flower	
2-Heptanone	110-43-0	C_7_H_14_O	1.9279	Fruity, slight medicinal fragrance	
Ethyl hexanoate	123-66-0	C_8_H_16_O_2_	1.3733	Sweet fruits	Esters
Methyleugenol	93-15-2	C_11_H_14_O_2_	1.3515	Cloves, cinnamon	Phenols
Benzothiazole	95-16-9	C_7_H_5_NS	1.2858	Rubber, sulfide	Others

## Data Availability

The original contributions presented in this study are included in the article. Further inquiries can be directed to the corresponding authors.

## Supplementary Materials

The following supporting information can be downloaded at https://www.mdpi.com/article/10.3390/foods15030560/s1. Table S1: Related information about *mokA-I*, *GADPH* primers; Table S2: RT-qPCR reaction system and condition; Figure S1: Permutation test graph of the OPLS-DA model.
