# Supplementary material for: Honeysuckle as a Bio-Enhancer in Monascus purpureus Fermentation: Synergistic Improvement of Monacolin K Yield and Flavor Complexity
Source: Foods. 2026 Feb 4;15(3):560. doi: 10.3390/foods15030560 (PMC12897098; doi:10.3390/foods15030560)
Supplement: Supplementary file 1 [file foods-15-00560-s001.zip › foods-4095195-supplementary.pdf]

Supplementary Tables

Table S1. Related information about *mokA-I*, GADPH primers

Table S2. RT-qPCR reaction system and condition

Figure S1. Permutation test graph of the OPLS-DA model

Table S1. Related information about *mokA-I*, GADPH primers

Genes	Primer pairs(5'-3')	length(bp)	Tm value
mokA	GACCTCGGTCATCTTGGC	18	59.1
	TTGTTCCAAGCGGTCTTC	18	57.3
mokB	AAACATCGTCACCAGTCT	18	50.7
	CTAAGTCGGGCATCTACC	18	53.1
mokC	CAAGCTGCGAAATACACCAAGCCTC	25	63.6
	AGCCGTGTGCCATTCCTTGTTGTCC	25	65.3
mokD	TTCATCTGCTGCTGGTAT	18	59.8
	AACTTCTCACCGTCAATG	18	58.7
mokE	ATCGCAGGTCACGCACATCCAAGTC	25	72.3
	GTAAAGGCAGCCCGAGCAGCTTCAT	25	71.1
mokF	GAGATCATAGTGGCCGACTGAA	22	59.8
	ACCGTCTCATCCAACCTCACGA	22	56.1
mokG	CCAGGTAACCAACGGATTA	19	56
	GATCAGAGCAGTCACCAG	18	52
mokH	CAGGAAATCTGGACTTACCCCATTG	25	65.8
	TGTTGGATTGTTGTTGGAGATATAC	25	59.2
mokI	ATGTTGAATGGCAATGATGG	20	60.9
	CAGCGTGGGTGATGTATC	18	61.7
GADPH	CCGTATTGTCTTCCGTAAC	19	55.4
	GTGGGTGCTGTCATACTTG	19	57.6

Table S2. RT-qPCR reaction system and condition

Reagents	Dosage(10uL)	Reaction condition
RNase-free ddH_2O	10	Three-step procedure: pre-denaturation at 95°C for 15 min; 95°C for 10 s, 60°C for 20 s, 72°C for 30 s, GOTO 2, 40X; melting curve analysis, 65°C-95°C, increments of 0.5°C for 5 s. The melting curve was analyzed at 0.5°C for 5 s and at 0.5°C for 5 s. The melting curve was then analyzed at 0.5°C.
cDNA template	0.6	
forward primer	0.6	
reverse primer	1	
2xSuperReal PreMix Plus	7.8	

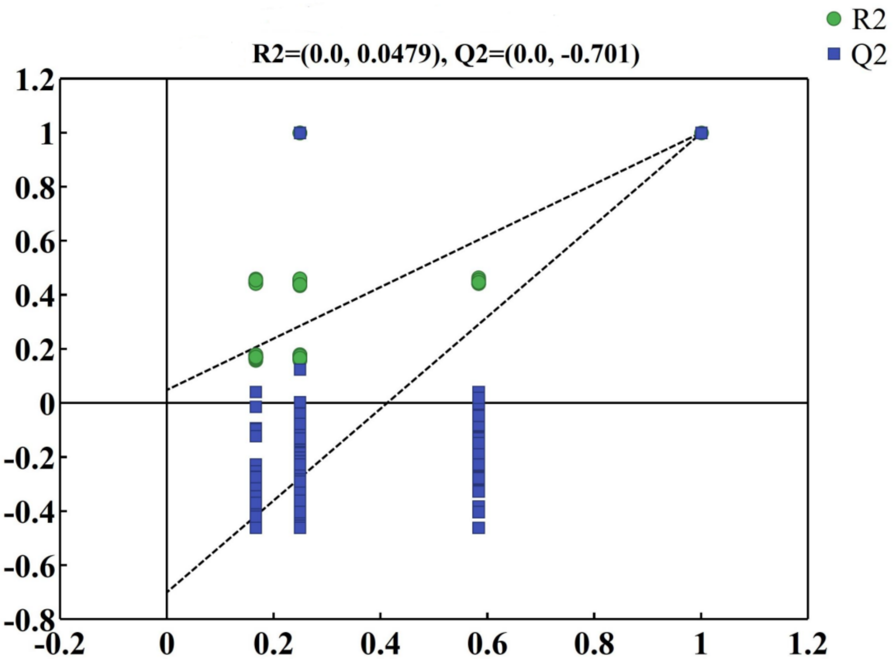

Figure S1. Permutation test graph of the OPLS-DA model.
